# Nomogram to predict collapse-free survival after core decompression of nontraumatic osteonecrosis of the femoral head

**DOI:** 10.1186/s13018-021-02664-3

**Published:** 2021-08-21

**Authors:** En-Ze Zhao, Zun-Han Liu, Wei-Nan Zeng, Zi-Chuan Ding, Zhen-Yu Luo, Zong-Ke Zhou

**Affiliations:** 1 Department of Orthopedics, Orthopedic Research Institute, West China Hospital/West China School of Medicine, Sichuan University, 37# Wuhou Guoxue Road, Chengdu, People’s Republic of China; 2 Department of Orthopedics, Chongqing General Hospital, University of Chinese Academy of Sciences, Chongqing, 400014 China

**Keywords:** Core decompression, Risk factors, Nomogram, Total hip arthroplasty

## Abstract

**Background:**

Nontraumatic osteonecrosis of the femoral head (NONFH) is a devastating disease, and the risk factors associated with progression into collapse after core decompression (CD) remain poorly defined. Therefore, we aim to define risk factors associated with collapse-free survival (CFS) after CD of precollapse NONFH and to propose a nomogram for individual risk prediction.

**Methods:**

According to the baseline characteristics, clinical information, radiographic evaluations, and laboratory examination, a nomogram was developed using a single institutional cohort of patients who received multiple drilling for precollapse NONFH between January 2007 and December 2019 to predict CFS after CD of precollapse NONFH. Furthermore, we used *C* statistics, calibration plot, and Kaplan-Meier curve to test the discriminative ability and calibration of the nomogram to predict CFS.

**Results:**

One hundred and seventy-three patients who underwent CD for precollapse NONFH were retrospectively screened and included in the present study. Using a multiple Cox regression to identify relevant risk factors, the following risk factors were incorporated in the prediction of CFS: acute onset of symptom (HR, 2.78; 95% CI, 1.03–7.48; *P* = 0.043), necrotic location of Japanese Investigation Committee (JIC) C1 and C2 (HR, 3.67; 95% CI, 1.20–11.27; *P* = 0.023), necrotic angle in the range of 250–299°(HR, 5.08; 95% CI, 1.73–14.93; *P* = 0.003) and > 299° (HR, 9.96; 95% CI, 3.23–30.70; *P* < 0.001), and bone marrow edema (BME) before CD (HR, 2.03; 95% CI, 1.02-4.02; *P* = 0.042). The *C* statistics was 0.82 for CFS which revealed good discriminative ability and calibration of the nomogram.

**Conclusions:**

Independent predictors of progression into collapse after CD for precollapse NONFH were identified to develop a nomogram predicting CFS. In addition, the nomogram could divide precollapse NONFH patients into prognosis groups and performed well in internal validation.

## Introduction

Osteonecrosis of the femoral head (ONFH) is a devastating disease and becomes an increasing worldwide health problem [1, 2]. More than 70% of ONFH is caused by nontraumatic factors, known as nontraumatic ONFH (NONFH), and it typically affects a relatively young age group [3]. Unfortunately, NONFH often leads to femoral head collapse which inevitably results in hip arthroplasty later. Despite the 10-year survivorship of total hip arthroplasty (THA) was tremendously improved with the development of new techniques, patients in such a young age group still likely receive revision surgery in their lifetime [4]. To prolong the time interval before progression into collapse even to avoid collapse, core decompression (CD) was proposed to preserve the affected hips by reducing intraosseous hypertension and promoting revascularization of the femoral head, which have been reported with clinical success especially in precollapse NONFH cases [5–7].

However, NONFH is an intractable disease, with up to 24.6 to 42.8% of patients experiencing collapse after CD [8–10]. Therefore, it is essential to target patients who are most likely to benefit from this intervention according to the prediction of the prognosis. Accurate prediction of prognosis is the key to determine the frequency of the follow-up and adjuvant therapy and to balance patient expectations with useful information about the short-term and long-term outcomes for precollapse NONFH patients after CD. Despite several radiomic features associated with the collapse that have been reported in previous studies [11, 12], reliable prognostication among patients with precollapse NONFH after CD remains a challenge.

Although the Ficat and Arlet system has historically been the most frequently applied classification [13], its stratification systems may not be suitable to determine the prognosis of individual patients. Further, to our knowledge, few studies in the literature have proposed a nomogram to predict the collapse-free survival (CFS) after CD in precollapse NONFH patients. Therefore, the purpose of the present study was to identify the risk factors of progression into collapse after CD in precollapse NONFH patients. Further, we proposed to develop a nomogram and internally validate it to predict the individual risk of CFS after CD in precollapse NONFH patients.

## Materials and methods

A total of 338 patients were diagnosed with NONFH and underwent multiple drilling in our institution from January 2007 to December 2019. The inclusion criteria were as follows: (1) patients with NONFH aged > 18 and < 70 years; (2) patients who received multiple drilling on one hip and THA on the other side simultaneously; (3) unilateral hip was classified as stage I or IIA according to the Ficat and Arlet system determined by preoperative X-ray and magnetic resonance imaging (MRI) before receiving CD. Exclusion criteria were as follows: (1) patients who were followed for less than three years; (2) patients who underwent operative intervention for the purpose of preserving the femoral head before and after the index surgery; (3) patients without MRI data before CD; (4) patients experiencing femoral neck fracture or femoral intertrochanteric fracture during the follow-up; (5) patients were lost and could not be contacted. Based on the inclusion and exclusion criteria, 173 eligible patients were retrospectively screened and included in this study.

### Surgical technique

The surgical procedure of CD was conducted as described by Mont et al [6]. All procedures were performed under general anesthesia and patients were placed in a supine position using a straight incision skin incision created from the tip of the greater trochanter. Under the guidance of C-arm fluoroscopy, a guide pin was inserted into the lateral cortex of the femur and drilling through the proximal part of the femoral neck towards the center of the femoral head with appropriate depth and alignment. Then, drill channels were created with cannulated drill bit until reaching 5 mm beneath the subchondral bone. Similarly, another one or two additional channels were drilled toward the necrotic lesions to reduce intraosseous hypertension. Due to mild inflammatory reaction, autogenous bone grafting was widely used as an auxiliary procedure which could provide short-term structural support after core decompression. Therefore, 68 patients received CD and autogenous bone-grafting with cancellous bone harvested from the contralateral femoral head after underwent THA.

### Clinical and radiographic evaluations

All demographic data were collected including age, gender, and body mass index (BMI). Clinical evaluations were conducted preoperatively and annually thereafter until the final follow-up. Patients with unusual symptoms or abnormal radiographic findings were evaluated more frequently. Clinical evaluations of the precollapse hips were performed including the etiology of the NONFH, classification system of the Association Research Circulation Osseous (ARCO) and the Ficat and Arlet, and the time span between two hips starting presence of symptom [14]. Symptomatic hips were defined as hips with 30 or fewer points (mild pain, no effect on average activities, rare moderate pain with unusual activity) before CD based on the pain domain score in the Harris hip score [15]. According to the median of the time span between two hips starting presence of symptom before CD, we divided the hips receiving CD into (1) asymptomatic, (2) acute onset of symptom (<10 months), and (3) delayed onset of symptom (≥ 10 months). Since there have been reports of several biomarkers that may become risk factors such as inherited thrombophilia and hypofibrinolysis in osteonecrosis development [16, 17], we also attempted to use laboratory examination to predict the prognosis of CD. Therefore, the blood examination before CD including triglyceride, total cholesterol, white blood cell (WBC) count, hemoglobin level, platelet count, and antithrombin III level were also recorded [18].

Radiographic evaluations of MRI parameters and X-ray were conducted for patients preoperatively and at each follow-up. The precollapse of NONFH was defined as the stage of Ficat I or IIA and collapse was defined as the presence of femoral head depression > 2 mm according to radiographs [19]. Lesion location was assessed on midcoronal MRI using Japanese Investigation Committee (JIC) classification [20]. The necrotic angle was estimated by calculating the sum of the necrotic angle on coronal and sagittal MRI images according to the modified Kerboul method [21]. We divided the necrotic angle into four categories: grade 1 (< 200°), grade 2 (200–249°), grade 3 (250–299°), and grade 4 (> 299°). We also recorded bone marrow edema (BME) before CD which was defined as the presence of diffuse and low-signal intensity area on T1 weighted images with high-signal intensity on fat-suppressed T2 weighted images beyond the necrotic lesion [22]. The primary outcome of interest was the CFS based on the maintained spherical shape of the femoral head in the radiographic image (femoral head depression < 2 mm).

### Statistical analysis

Continuous variables were recorded with mean ± standard deviation and were analyzed using the Student’s *t*-test or the Wilcoxon rank-sum test. Categorical values were recorded as whole numbers and were analyzed using the chi-squared or Fisher’s exact test. To identify the risk factors for progression into collapse after CD, the univariate Cox proportional hazards regression model was used. Covariates with a *P* value < 0.1 were integrated into the backward stepwise multivariate Cox proportional hazards regression model, where variables with *P* < 0.05 were considered possible predictors. Hazard ratios (HRs) and 95% CIs were reported. Selected variables were incorporated in the nomogram to predict the probability of CFS rate after CD of precollapse NONFH using statistical software (R, version 4.0.2; http://www.r-project.org). The regression coefficients were used to allocate points in the nomogram. The CFS for the cohort was assessed with the Kaplan-Meier method, and the difference in the CFS was tested using the log-rank test.

The discriminating ability and calibration were used to assess the model performance. *C* statistics was used to assess the performance of the nomogram described by Harrell et al [23]. A calibration plot with a bootstrapped sample of the study cohort was used to assess the calibration of the model. To further evaluate the calibration of the model, Kaplan-Meier curves were plotted over the tertiles of patients stratified based on the scores predicted by the nomogram. To quantify overfitting, the model was confirmed with bootstrapped resampling. All statistical analyses were performed using software programs (SPSS, version 25.0, IBM; and R, version 4.0.2, http://www.r-project.org). *P* values < 0.05 were considered statistically significant.

## Results

In total, the mean age of all included patients was 43.2 years and 13.3% of the patients were female. The mean BMI of all patients was 24.1 kg/m^2^. NONFH was associated with alcohol abuse in 113 patients, steroid therapy in 27 patients and the remaining 33 was idiopathic NONFH. According to the Ficat classification for NONFH, 18 patients were identified as Ficat I stage and 155 patients were identified as Ficat IIA stage at the time of surgery. Based on ARCO classification, there were 8 cases of ARCO IA, 9 cases of ARCO IB, 1 case of ARCO IC, 67 cases of ARCO IIA, 51 cases of ARCO IIB, and 37 cases of ARCO IIC at the time of surgery. Of all patients in this study, 31 patients received bisphosphonates and 125 patients received vitamin D or calcium tablet after CD. The mean duration of follow-up was 53.9 months. At 36 months, 23 patients experienced collapse after CD and the 3-year CFS was 85.9% (95% CI, 80.6–91.1%). To further display the feature of the cohort, we divided the cohort as collapse and survival groups based on the primary outcome (Table 1).

**Table 1:** Baseline characteristics of all recruited patients

Variable	Collapse group (*n* = 44)	Survival group (*n* = 129)	*p* value
Age (years)	43.3 ± 7.4	43.2 ± 8.7	0.965
Gender (male/female)	38/6	112/17	0.938
Body mass index (kg/m^2^)	24.5 ± 4.5	24 ± 3.7	0.512
Etiology			0.312
Alcohol abuse	31	82	
Corticosteroid use	8	19	
Idiopathic	5	28	
Ficat classification			0.741
Stage I	4	14	
Stage IIA	40	115	
ARCO classification			< .001
IA	2	6	
IB	2	7	
IC	0	1	
IIA	4	63	
IIB	10	41	
IIC	26	11	
Symptom			< .001
Acute onset of symptom	14	23	
Asymptomatic	24	79	
Delayed onset of symptom	6	27	
Necrotic location			< .001
A	1	22	
B	3	45	
C1	15	51	
C2	25	11	
Necrotic angle			< .001
< 200°	5	74	
200–249°	10	36	
250–299°	18	12	
> 299°	11	7	
BME before CD			< .001
No	29	116	
Yes	15	13	
Bone grafting (no. of hips)			0.542
No	25	80	
Yes	19	49	
Triglyceride	2.05 ± 1.16	1.95 ± 1.17	0.611
Total cholesterol	4.50 ± 0.80	4.66 ± 0.92	0.305
White blood cell	6.6 ± 2	6.7 ± 1.9	0.857
Hemoglobin	138.0 ± 12.1	140.0 ± 16.8	0.493
Platelet	188.8 ± 53.5	186.3 ± 49.9	0.787
Antithrombin III	93 ± 11.9	92.5 ± 10.7	0.789

*BME*, bone marrow edema; *CD*, core decompression; *ARCO*, Association Research Circulation Osseous classification

Risk factors with *P* < 0.05 in univariate Cox regression were selected as candidate variables for the prediction model including symptom, necrotic location, necrotic angle, BME before CD, and ARCO classification. Backward stepwise selection in the multivariable Cox proportional hazards regression modeling identified the following 4 variables that had the strongest association with collapse risk: acute onset of symptom (HR, 2.78; 95% CI, 1.03–7.48; *P* = 0.043), necrotic location of JIC C1 and C2 (HR, 3.67; 95% CI, 1.20–11.27; *P* = 0.023), necrotic angle in the range of 250–299°(HR, 5.08; 95% CI, 1.73–14.93; *P* = 0.003), and > 299° (HR, 9.96; 95% CI, 3.23–30.70; *P* < 0.001), BME before CD (HR, 2.03; 95% CI, 1.02–4.02; *P* = 0.042) (Table 2).

**Table 2:** Cox proportional hazards regression model showing the association of variables with collapse-free survival

Variable	Univariate analysis		Multivariate analysis	
	HR (95% CI)	*P* value	HR (95% CI)	*P* value
Factors selected				
Symptom				
Delayed onset of symptom	1 (reference)	NA	1 (reference)	NA
Acute onset of symptom	3.28(1.26–8.58)	0.015	2.78(1.03–7.48)	0.043
Asymptomatic	1.73(0.70–4.25)	0.230	2.26(0.918–5.58)	0.076
Necrotic location				
A/B	1 (reference)	NA	1 (reference)	NA
C1/C2	8.94(3.20–25.03)	< 0.001	3.67(1.20–11.27)	0.023
Necrotic angle				
< 200°	1 (reference)	NA	1 (reference)	NA
200–249°	3.62(1.24–10.62)	0.019	2.67(0.87–8.25)	0.087
250–299°	11.62(4.30–31.37)	< 0.001	5.08(1.73–14.93)	0.003
> 299°	16.36(5.64–47.48)	< 0.001	9.96(3.23–30.70)	< 0.001
BME before CD				
No	1 (reference)	NA	1 (reference)	NA
Yes	3.09(1.65–5.77)	< 0.001	2.03(1.02–4.02)	0.042
Factors not selected				
Age (years)	1.00(0.96–1.03)	0.913	NA	NA
Gender				
Female	1 (reference)	NA	NA	NA
Male	0.81(0.34–1.91)	0.626	NA	NA
Body mass index (kg/m^2^)	1.04(0.97–1.13)	0.285	NA	NA
Etiology				
Alcohol abuse	1 (reference)	NA	NA	NA
Corticosteroid use	1.17(0.54–2.54)	0.700	NA	NA
Idiopathic	0.67(0.26–1.72)	0.401	NA	NA
Ficat classification				
Stage I	1 (reference)	NA	NA	NA
Stage IIA	1.27(0.45–3.55)	0.649	NA	NA
ARCO classification				
IA	0.32(0.08–1.34)	0.117	NA	NA
IB	0.20(0.05–0.86)	0.031	NA	NA
IC	0.00(0.00–0.00)	0.977	NA	NA
IIA	0.06(0.02–0.17)	< 0.001	NA	NA
IIB	0.23(0.11–0.48)	< 0.001	NA	NA
IIC	1 (reference)	NA	NA	NA
Bone grafting (no. of hips)				
No	1 (reference)	NA	NA	NA
Yes	0.65(0.35–1.22)	0.177	NA	NA
Triglyceride	1.08(0.85–1.37)	0.536	NA	NA
Total cholesterol	0.84(0.60–1.17)	0.304	NA	NA
White blood cell	0.97(0.83–1.14)	0.699	NA	NA
Hemoglobin	0.99(0.98–1.01)	0.425	NA	NA
Platelet	1.00(0.99–1.01)	0.642	NA	NA
Antithrombin III	1.00(0.98–1.03)	0.852	NA	NA

*BME*, bone marrow edema; *CD*, core decompression; *ARCO*, Association Research Circulation Osseous classification; *HR*, hazard ratio; *NA*, not applicable

**Fig. 1:**
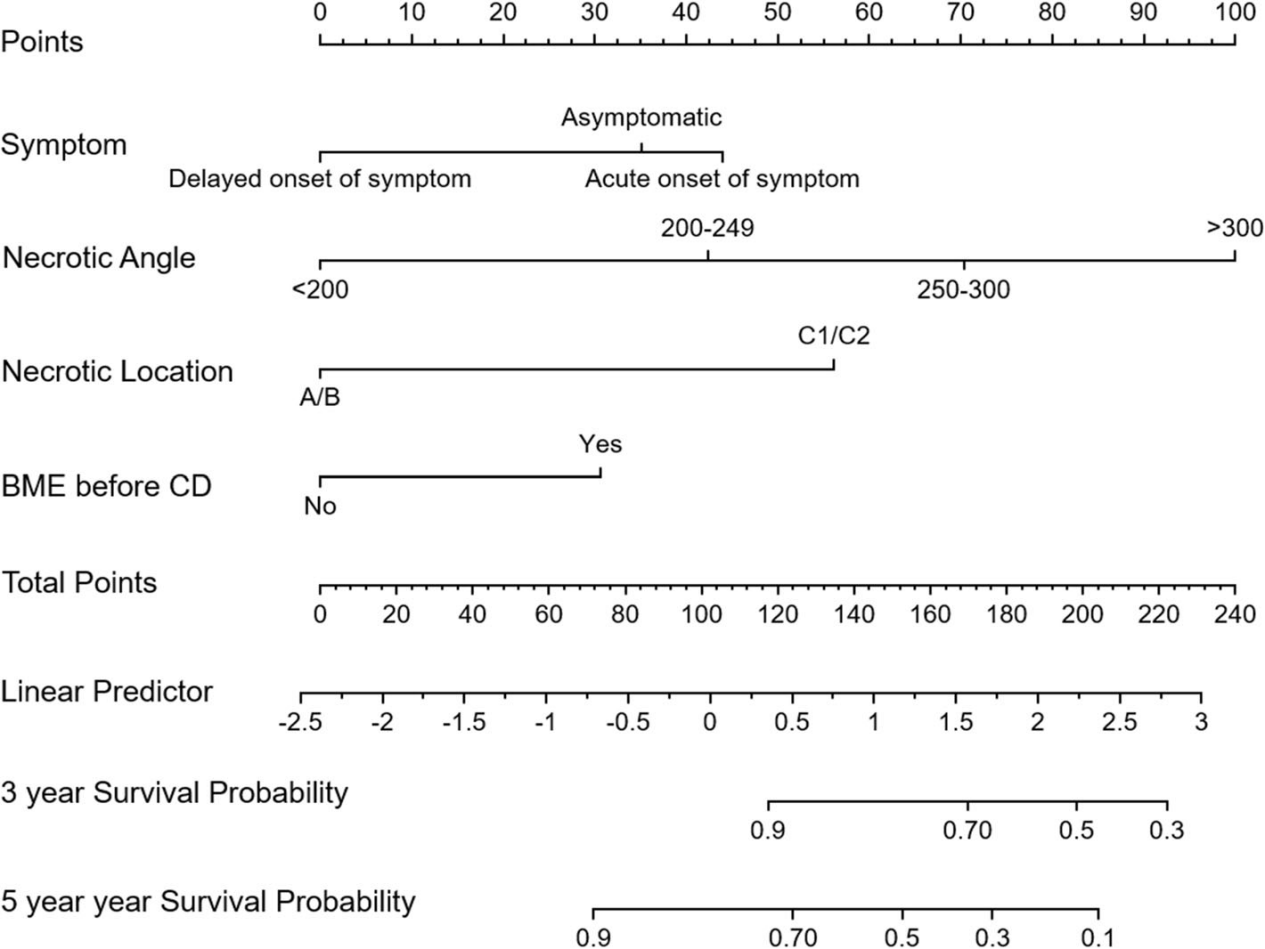
Nomogram predicting collapse-free survival in patients after core decompression

**Fig. 2:**
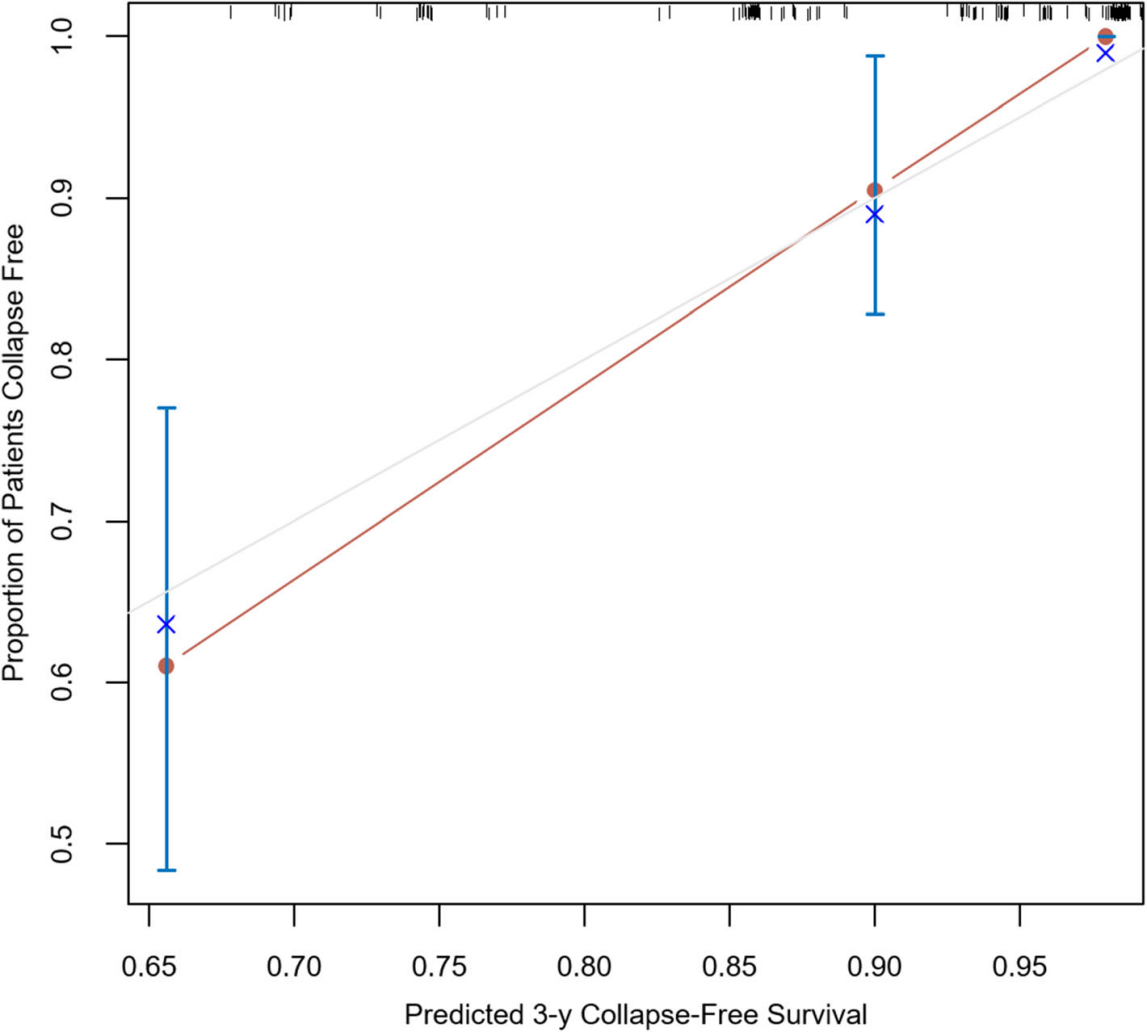
Calibration plot comparing predicted and actual collapse-free survival probabilities at 3-year follow-up. The 50-sample bootstrapped calibration plot for the prediction of 3-year collapse-free survival is shown. The gray line represents the ideal fit; circles represent nomogram-predicted probabilities; the cross represents the bootstrap-corrected estimates; and the error bars represent the 95% CIs of these estimates

**Fig. 3:**
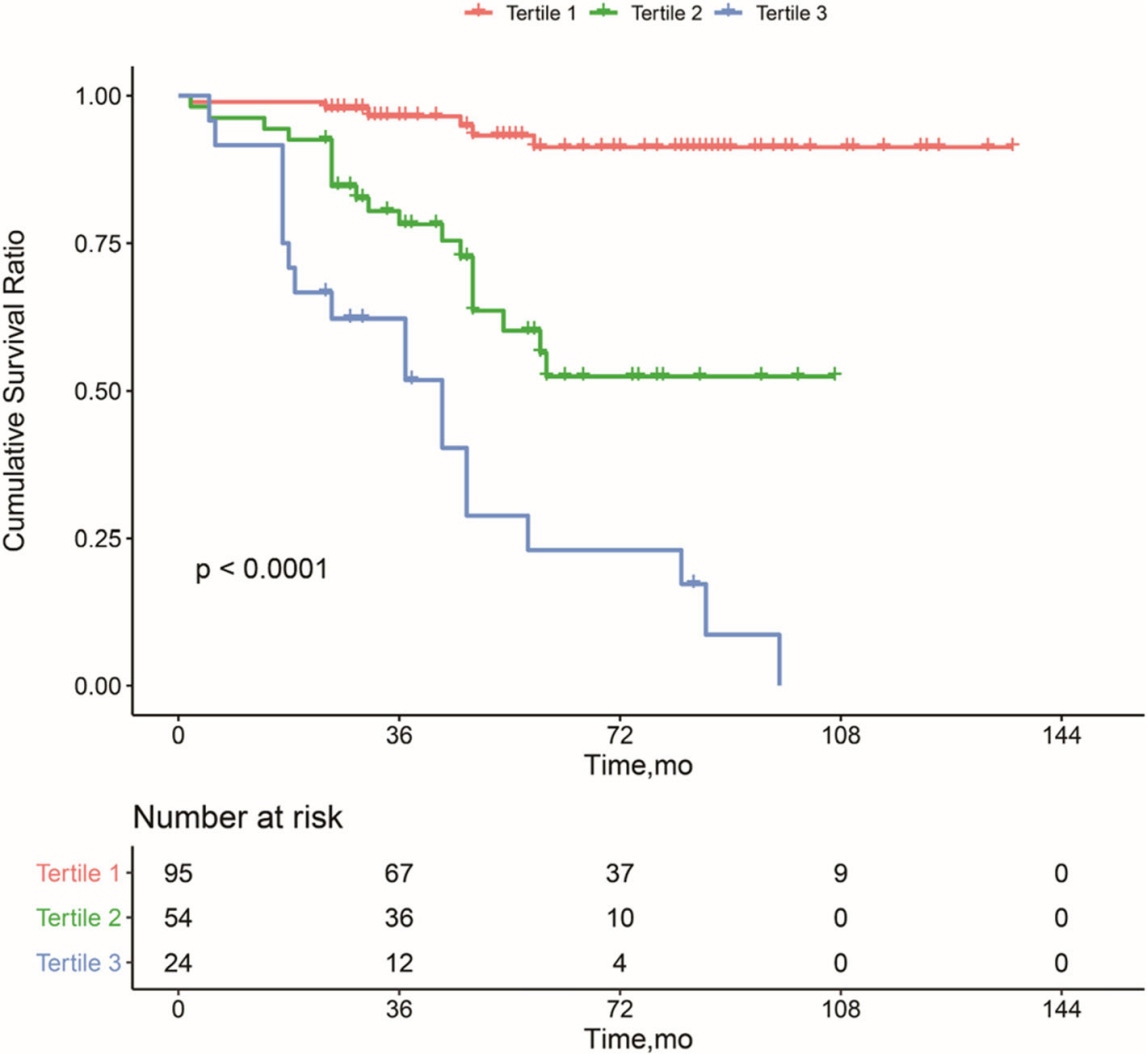
Kaplan-Meier curves demonstrating survival in patients after core decompression for precollapse nontraumatic osteonecrosis of the femoral head according to tertiles of predicted survival

The nomogram to predict CFS of the NONFH patients after CD is shown in Fig. 1. There was an association between worse prognosis and higher total points according to the sum of the allocated number of points for each factor in the nomogram. The accuracy of the model and potential model overfit were evaluated by 1000 resampled bootstrap validation. The 50-sample bootstrapped calibration plot for the prediction of CFS is shown in Fig. 2. The discriminative ability of the proposed model for CFS after CD was evaluated using the *C* statistics (0.82). Kaplan-Meier curves were also plotted based on the predicted probability of stratified by the tertile of the 5-year CFS predicted probability calculated from the nomogram to further evaluate the discriminative ability of the proposed model (Fig. 3). Compared with patients in tertiles 1 (96.6% for 3-year CFS and 91.3% for 5-year CFS) and tertiles 2 (78.2% for 3-year CFS and 52.4% for 5-year CFS), those with the lowest predicted CFS (tertile 3) had significantly worse outcomes (62.2% for 3-year CFS and 23.0% for 5-year CFS) (log rank, *P* < .001).

## Discussion

While CD was recommended as the first surgical therapeutic option in precollapse NONFH [24], 24.6 to 42.8% of the patients still experienced progression to collapse after CD reflecting the prognostic heterogeneity associated with the disease which was dependent on numerous factors [8–10]. It is necessary to distinguish which patients are most likely to benefit from CD based on the prediction of prognosis. Accurate prognostication is of significance which could not only help to determine further treatment plans but also enable patients to actively cooperate with surgeons through enhancing patients’ cognition about their outcome. The Ficat and Arlet system is the most commonly used for classification and prognosis, but the prognostic factors associated with collapse are diverse and an individualized method for risk stratification of the patients with precollapse NONFH after CD remains unclear [2]. Therefore, we identified independent predictors and developed a nomogram to predict an individual’s CFS after CD for precollapse NONFH with rigorous evaluation and internal validation, which had rarely been reported in the literature.

Although most orthopedic surgeons agree that CD should be taken to slow progression and to prevent THA in precollapse NONFH, there was no consensus regarding the optimal CD technique including multiple drilling and CD with tantalum rod placement, vascularized and nonvascularized bone graft [25–27]. While standard CD was the most widespread joint-preserving procedure proposed in the recent decade, Al Omran [28] reported that no differences were found in the outcome or complication rate between patients who underwent standard CD and multiple drilling at a mean 3-year follow-up. In our cohort, multiple drilling was used in all included patients of precollapse NONFH with relative better outcomes than prior studies [29, 30]. A possible interpretation might include the following: first, multiple drilling with small diameter pin could easily reach the anterior portion of the femoral head reducing intraosseous hypertension; second, the technique was one of the mini-invasive interventions which could reduce the possibility of penetrating the femoral head and damaging the articular cartilage; third, due to the small diameters of drilling, the procedure provided mechanical support, retained the anatomic structure of the femoral head, and reduced the risk of subtrochanteric fracture. In the present study, we established a nomogram to predict 3-year CFS after CD in patients with precollapse NONFH. Since Bradway et al. [31] reported a series of natural history with 47% of hips going on to collapse in less than 1 year and 80% of hips progressing to collapse within 3 years, the prognostication of 3-year CFS in the present study had clinical significance. In addition, the proposed nomogram was able to identify distinct groups of the patients who were at different risks of collapse when stratified into tertiles. Most importantly, the nomogram presented good discriminative ability with a *C* index of 0.82 for predicting CFS. Collectively, the nomogram could provide patient-specific information on the risk of collapse for patients with precollapse NONFH after CD.

We agreed the finding of the previous reports that lesion size was the most important factor to predict the outcomes of patients with precollapse NONFH after CD [12, 32, 33]. Several classification systems have been proposed to categorize and quantify NONFH on the lesion size such as the Steinberg classification and the modified Kerboul method [21, 34]. The Steinberg classification was based on lesion volume while the modified Kerboul method was proposed based on the sum of the arc of the necrotic lesion on both the midcoronal and the midsagittal MRI. Although the Steinberg classification using volumetric measurement seemed more precise than the modified Kerboul method, previous studies have suggested that the modified Kerboul method was more acceptable than the Steinberg classification because it was convenient to use whereas the Steinberg classification was time-consuming if calculation software could not be used [11, 35]. In addition, the modified Kerboul method was more accurate than several methods of measurement in a single plane in quantifying the lesion size. Therefore, given the clinical practicability, we applied the modified Kerboul method in the present study to quantify the lesion size for predicting the prognosis of CD.

As far as the location of the lesion was concerned, quantitative analysis of necrotic lesion morphology suggested that the location of necrotic lesions relative to the acetabular weight-bearing portion was a significant prognostic factor of collapse even if the necrotic size was small [36, 37]. Therefore, the location and size of necrotic lesions were considered independently relative factors of collapse. Furthermore, a prior study reported that Japanese Investigation Committee (JIC) classification was a reliable and effective method to distinguish the location of necrotic lesions especially for early-stage NONFH, and only 3% of hips of JIC types A and B had progressed to collapse during a 9-year follow-up in a prior study [38]. Therefore, we divided hips into JIC type A/B and JIC type C/D to conduct the development of the nomogram. In addition, it was known that BME was a characteristic MRI presence associated with postcollapse NONFH [39–41]. However, Hatanaka et al. reported that BME might be a sign of occult fracture in precollapse NONFH patients and the Kaplan-Meier survivorship analysis showed a significant difference in the survival rate between the BME positive and negative precollapse NONFH cases [42]. As a result, BME was included as a variable in the proposed nomogram.

One particular strength of the present study was that it took into account not only radiographic variables but also a wide array of other variables previously reported to be associated with the prognosis of precollapse NONFH. It was known that precollapse NONFH was typically discovered in the contralateral side of a symptomatic collapsed hip (hip or groin pain), then the precollapse hip might occur over time. In the present study, we found an association between the time span of two hips starting presence of symptom and collapse after receiving CD, indicating that patients who have acute symptomatic onset of the precollapse hip might have a more easily progressive form of the disease, which was also confirmed by a previous study [43]. As for asymptomatic precollapse hip, Hungerford et al. reported that 73% of asymptomatic precollapse hips had progressed to collapse at a mean 11-year follow-up and recommended prophylactic hip-preserving surgical treatment of asymptomatic hips, regardless of the lesion size or location [44]. In the present study, we found 24% of asymptomatic precollapse NONFH patients experienced collapse after CD, which further confirmed the positive effect of CD. We also recorded hematological indicators before the surgery as a previous study found that aberrant lipid metabolism and coagulation abnormalities might have a correlation with NONFH [16, 17]. However, these hematological indicators could not be included as prognostic factors in the present study. A possible interpretation might be that the sample size was still small limiting the analysis and the included patients might not be that representative.

Several limitations exist in the present study. Firstly, the nomogram was based on a single-center retrospective study that could limit its applicability to other populations.

Secondly, the sample size was still small. As such, some analyses might have been limited. Furthermore, the data on certain factors, such as the exact dose of corticosteroid administration of some included patients, were unavailable; therefore, their effect or potential incorporation in the nomogram could not be evaluated. Thirdly, although our nomogram was internally validated using bootstrap validation, future studies are needed to externally validate the proposed nomogram.

## Conclusions

Using a relatively large single-center data set of patients who underwent CD for precollapse NONFH, several independent prognostic variables were identified to predict CFS in the present study. We proposed a nomogram and carefully assessed the model which provided satisfactory accuracy for predicting postoperative outcomes in internal validation and stratified patients into different prognostic groups regarding the collapse of the femoral head. External validation is necessary in future studies to confirm the value of the proposed nomogram in predicting the prognosis after CD for precollapse NONFH.

## Data Availability

The datasets analyzed during the current study are available from the corresponding author on reasonable request.

## Abbreviations

NONFH: Nontraumatic osteonecrosis of the femoral head
CD: Core decompression
CFS: Collapse-free survival
ONFH: Osteonecrosis of the femoral head
THA: Total hip arthroplasty
BME: Bone marrow edema
JIC: Japanese Investigation Committee
HR: Hazard ratio
NA: Not applicable
